# The Positive Modulation Effect of a 6-Week Consumption of an Anthocyanin-Rich Mulberry Milk on Working Memory, Cholinergic, and Monoaminergic Functions in Healthy Working-Age Adults

**DOI:** 10.1155/2021/5520059

**Published:** 2021-08-31

**Authors:** Wipawee Thukham-mee, Jintanaporn Wattanathorn, Pongsatorn Paholpak, Poonsri Ransikachi, Nawanant Piyavhatkul

**Affiliations:** ^1^Research Institute for High Human Performance and Heath Promotion, Khon Kaen University, Khon Kaen 40000, Thailand; ^2^Department of Physiology, Faculty of Medicine, Khon Kaen University, Khon Kaen 40000, Thailand; ^3^Department of Psychiatry, Faculty of Medicine, Khon Kaen University, Khon Kaen 40000, Thailand

## Abstract

Due to the increase of stress-related memory impairment accompanying with the COVID-19 pandemic and financial crisis, the prevention of cognitive decline induced by stress has gained much attention. Based on the evidence that an anthocyanin-rich mulberry milk demonstrated the cognitive enhancing effect, we hypothesized that it should be able to enhance memory in working-age volunteers who are exposed to working stress. This study is an open-label, two-arm randomized study. Both men and women volunteers at age between 18 and 60 years old were randomly assigned to consume the tested product either 1 or 2 servings daily for 6 weeks. All subjects were assessed for cortisol, acetylcholinesterase (AChE), monoamine oxidase (MAO), monoamine oxidase type A (MAO-A), and monoamine oxidase type B (MAO-B) in saliva, and their working memory was determined both at baseline and at a 6-week period. The results showed that the working memory of subjects in both groups was enhanced at the end of the study period together with the reduction of saliva cortisol. The suppression of AChE, MAO, and MAO-A was also observed in subjects who consumed the tested product 2 servings daily. Therefore, we suggest the memory enhancing effect of an anthocyanin-rich mulberry milk. The possible mechanism may occur primarily via the suppression of cortisol. In addition, the high dose of mulberry milk also suppresses AChE, MAO, and MAO-A.

## 1. Introduction

Stress, an inevitable event nowadays, is continually rising worldwide. Living in the era of the COVID-19 pandemic and financial crisis gives rise to the unintended adverse effects of stress including the increase risk of stress-related memory impairment [1, 2]. Stress activates the hypothalamic-pituitary-adrenocortical (HPA) axis which in turn gives rise to the release of glucocorticoids especially cortisol or stress hormone and results in memory deficit [3]. Due to the important roles of working memory on a daily life activity and on career success together with the increasing stress prevalence, the prevention of stress-related cognitive impairment has gained much attention.

Mulberry or *Morus alba*, a plant in the family of Moraceae, has been widely planted in Thailand. The ripe fruit of mulberry has been consumed both as a fruit and as medicine. Our previous studies have demonstrated that it possesses antioxidant, anti-inflammation neuroprotection, and memory enhancer [4–7]. Recently, we have demonstrated the clinical evidence that within 1.5 hours after the consumption of an anthocyanin-rich mulberry milk which contains mulberry fruit 10 g, the children at the age between 6 and 12 years old show the reduction of saliva cortisol together with the memory enhancement [8]. Based on this information, we hypothesized that a 6-week consumption of an anthocyanins-rich mulberry milk should be able to decrease stress hormone and improve working memory in healthy working-age volunteers. Due to the lack of available data concerning this point, this study is set up to test this hypothesis.

## 2. Materials and Methods

### 2.1. Anthocyanin-Rich Mulberry Milk

Anthocyanin-rich mulberry milk (Memberry®) used in this study was kindly supported by Mark One Innovation Center Company Limited of MK Restaurant Group. Each serving (180 ml) contains total phenolic compounds, flavonoids, and anthocyanins at the concentrations of 1415.17 ± 1.85/mg *μ*g GAE, 667 ± 1.07 *μ*g quercetin/mg, and 34.30 ± 0.74 mg cyanidin glucoside equivalent/L, respectively.

### 2.2. Study Design

This open-label, two-arm randomized study was designed to collect data on the potential effect of anthocyanin-rich mulberry milk consumption for 6 weeks on working memory in healthy working-age adults. The possible underlying mechanisms were also explored. It was performed according to the Declaration of Helsinki (ethical principles for research involving human subjects), and all procedures were under the approval of the Institute Human Ethical Committee under HE631051. This project was also registered online as part of the Thai Clinical Trial Registry (TCTR20201031002).

### 2.3. Participants and Interventions

A total of healthy 312 men and women at the age between 18 and 60 years old were recruited, and they were screened for eligibility. Only 300 subjects participated in this study. Individuals receiving treatment with any medications or herbs that may exert the influence on brain function or who had any prior diagnosis or history of stroke, heart disease, diabetes, gastrointestinal disease, cancer, central nervous system or psychiatric disorders, or traumatic brain injury were excluded. In addition, heavy smokers and alcohol addicts were also excluded. All participants provided written informed consent before participating in the study. Recruited subjects were randomly assigned to consume either one (180 ml) or two servings (360 ml) of mulberry milk daily for 6 weeks. All subjects still maintain their habitual diet and physical activity levels during the study period. They were given customized calendars and were asked to mark the days they missed consuming the assigned product. Unused study products were returned for compliance monitoring purposes.

**Figure 1:**
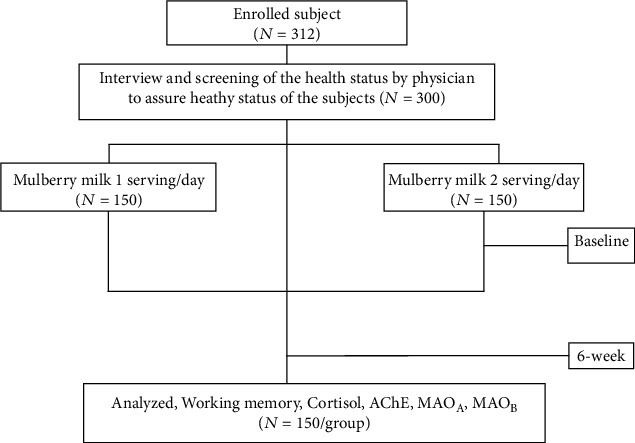
Flow diagram of subject distribution and study design.

Both primary and secondary outcomes of the subjects at baseline or before the intervention and at 6 weeks after the intervention were measured. The primary outcome in this study was working memory assessed by the computerized battery test consisting of word presentation, word recognition test, picture presentation, picture recognition, simple reaction time, digit vigilance, choice reaction time, and spatial working memory whereas the secondary outcomes were salivary cortisol, acetylcholinesterase, monoamine oxidase (MAO), monoamine oxidase type A (MAO_A_), and type B(MAO_B_). The schematic diagram illustrating the subject intervention procedure is shown in Figure 1.

### 2.4. Computerized Battery Test

The computerized battery test used in this study measured 4 domains of working memory comprising attention, continuity of attention, quality of memory, and speed of memory [9–11]. The test-retest reliability in each domain of working memory varied between 0.72 and 0.82 (power of attention = 0.75, continuity of attention = 0.78, quality of working memory = 0.82, and speed of memory = 0.72). The presentation of each task was performed via VGA color monitors. The responses of all tasks were recorded via a yes or no button box. The entire battery tests were around 20 minutes. The sequence of the test was performed as described below:

*Word presentation*: a subject was exposed with a 15-word presentation which was matched for frequency and concreteness. The stimulus duration was 1 s, as was the interstimulus interval. Both response and percentage of response accuracy were recorded.

*Picture presentation*: according to this test, each subject was exposed with a 20 photographic image exposure. The presentation was performed on the monitor at the rate of 1 every 3 s, with a stimulus duration of 1 s, for the participant to remember.

*Simple reaction time*: in this test, a 15-stimulus presentation was shown with an interstimulus interval that randomly varied between 1 and 3.5 s. All subjects were instructed to press the “yes” response button as quickly as possible every time the word “yes” was presented on the monitor. All response reaction times were recorded in milliseconds.

*Digit vigilance task*: each subject was exposed to 15 stimuli of digit presentation. The target digit was randomly selected and shown on the right side of the monitor screen, whereas the series of digit presentation was performed at the center of the screen at the rate of 80 min^−1^. All subjects were instructed to the “yes” button as quickly as possible every time the digit in the series matched the target digit.

*Choice reaction time*: all subjects were subjected to either the word “no” or the word “yes.” They were instructed to press the corresponding button as quickly as possible when the word presentation appeared on the monitor. Each set consisted of 50 trials. The stimulus word was randomly chosen with equal probability, with the interstimulus interval between 1 and 3.5 seconds.

*Spatial working memory*: according to this test, each subject was exposed to a targeted house picture with 9 windows. Four windows in this picture were illuminated. The subjects were instructed to memorize the position of the illuminated windows in the targeted house picture. Then, they were exposed to a series of house presentations with one illuminated window. The subject must match whether the illuminated window of each presented picture was shown at the location of the targeted house picture by pressing the “yes” or “no” response button as quickly as possible.

*Numeric working memory*: the subjects were exposed to 5 digits sequentially and they were instructed to memorize them. Following this process, a series of 30 probe digits were presented to the subjects and they must decide whether the probe digit was matched with the digits which previously exposed and must press the “yes” or “no” response button as quickly as possible.

### 2.5. Biochemical Assays

#### 2.5.1. Saliva Collection

Saliva samples were collected by using a Salivette collection device kit (No. 51.1534; Sarstedt, Numbrecht, Germany), according to the guideline. In brief, the sample was collected between 7.00 and 8.00 a.m. before breakfast. Each subject must rest for 1 min in a sitting position before saliva collection. Swab was directly placed into the subject's mouth under the tongue or allowed to move freely across the tongue. Do not place the swab between the cheek and gum and keep the swab in the mouth for two to three minutes to assure that the swab was completely saturated with enough volume (1/4 teaspoon was required). The samples were immediately frozen and transported to the laboratory for assays.

#### 2.5.2. Saliva Cortisol Assessment

The assessment of saliva cortisol was carried out by using ELISA kit. Briefly, 25 *μ*l of standard or saliva was mixed with 200 *μ*l of cortisol-HRP conjugate solution and incubated 37°C for 60 minutes. At the end of the incubation period, the solution was washed with buffer for three times and TMB (tetramethylbenzidine) substrate solution at the volume of 100 *μ*l was added and incubated at the room temperature for 15 minutes. Following this process, the stop solution was added. An absorbance at 450 nm was recorded via a microplate reader by using cortisol as a standard reference. The results were expressed as nanograms/milliliter.

#### 2.5.3. Determination of Acetylcholinesterase (AChE)

Saliva acetylcholinesterase (AChE) has been recognized as a valid indicator to indicate central cholinergic activity [12] so a saliva AChE was measured to explore the effect of anthocyanin-rich mulberry milk on the central cholinergic system. In brief, an aliquot of saliva or control at the volume of 10 *μ*l was mixed with the solution containing 10 *μ*l of 0.2 mM DTNB, 20 *μ*l of 0.1 mM PBS pH 8.0, and 10 *μ*l of 15 mM ATCI in the 96-well plates and subjected to a 5 minute-incubation period at room temperature. Following this step, an absorbance at 415 nm was recorded. The calculation of AChE activity was performed according to the following equation:

(1) $$\begin{array}{c} \text{AChE activity}=\left(\frac{∆A}{1.36\times 10^{4}}\right)\times \frac{1}{\left(20∕230\right)C}, \end{array}$$

where Δ*A* is the difference of absorbance/minute and *C* is protein concentration of brain homogenate.

### 2.6. Assessment of Monoamine Oxidase Enzyme Activity

Due to the correlation between saliva monoamine oxidase activity of MAO, MAO-A, and MAO-B and stress [13], the activities of the mentioned enzymes were also assessed in this study. The monoamine oxidase activity was determined by using the modified method of Holt and coworkers [14]. In brief, an aliquot of saliva at the volume of 50 *μ*l was mixed with a chromogenic solution consisting of 1 mM vanillic acid, 500 *μ*M 4-aminoantipyrine, and 4 U·ml peroxidase in 0.2 M potassium phosphate buffer, pH 7.6. After mixing thoroughly, tyramine which was used as substrate was added and incubated at 37°C for 30 minutes. At the end of an incubation period, the absorbance at 490 nm was recorded with a microplate reader (iMark™ Microplate Absorbance Reader). All data values were expressed as nanomoles/hour/milligram protein.

To measure the activity of MAO-A, each saliva sample at the volume of 50 *μ*l was transferred into a 96-well plate containing 50 *μ*l of 500 nM pargyline (MAO-A inhibitor) and subjected to a 30-minute incubation period at room temperature to allow the blocking of MAO-A activity by pargyline. Following this step, 200 *μ*l, 500 *μ*M tyramine was added to all wells. Then, the mixtures were incubated for 30 minutes at room temperature in the dark condition. According to this method, *p*-tyramine was used as substrate. At the end of incubation period, they were measured absorbance at the wavelength of 490 nm.

MAO-B activity was also determined by the same procedure but clorgyline was used as a substrate. The preparation of standard was also carried out as mentioned above but H_2_O_2_ was used to replace the saliva sample.

### 2.7. Statistical Analysis

All parameters were expressed as mean ± SEM. The statistical analysis was performed by using the Student paired *t*-test. The significance was considered when the *p* value was less than 0.05.

## 3. Results

### 3.1. Participant Flow and General Characteristics

A total of 300 healthy participants who met the study criteria from 312 enrolled subjects were randomly assigned to the 1 serving consumption (150) and 2 serving consumption (150) groups. No one dropped out from the study before the study completion as shown in Figure 1.

The general characteristics of subjects who consumed mulberry milk at dose of 1 serving and 2 serving per day are shown in Table 1. It was found that after 6 weeks of consumption, subjects who consumed mulberry milk 2 servings per day showed the significant elevation in pulse rate (*p* value < 0.05) and respiratory rate (*p* value < 0.01) whereas the subjects who consumed mulberry milk 1 serving per day showed the significant reduction in diastolic pressure (*p* value < 0.05). No other significant changes of any parameters were observed.

### 3.2. Effect on Working Memory

The effects of mulberry milk on working memory of subjects who consumed mulberry milk at a dose of 1 and 2 servings daily are shown in Tables 2 and 3. After a 6-week consumption period of mulberry milk, the subjects who consumed mulberry milk 1 and 2 servings per day showed the significant reduction of response time in word recognition, picture recognition, simple reaction, choice reaction time, spatial memory, and numeric working memory (*p* value < 0.001 all; *p* value < 0.001 and 0.01; *p* value < 0.001 all; *p* value < 0.05 and 0.01; *p* value < 0.001 all; and *p* value < 0.001 all). In addition, they also showed the significant increase in %accuracy in word recognition, picture recognition, digit vigilance, and spatial time (*p* value < 0.001 all; *p* value < 0.001 all; *p* value < 0.05 and 0.01; and *p* value < 0.01 all).

### 3.3. Biochemical Assays

Tables 4 and 5 showed the effect of mulberry milk on saliva cortisol and on the changes of salivary changes of AChE, MAO, MAO-A, and MAO-B. It was found that only saliva cortisol showed the significant reduction in subjects who consumed mulberry milk 1 serving per day (*p* value < 0.001). However, subjects who consumed mulberry milk at a dose of 2 servings per day significantly showed to have decreased cortisol, AChE, MAO, and MAO-A in saliva (*p* value < 0.001, 0.05, 0.05, and 0.05, respectively).

## 4. Discussion

In the study of cognitive function, especially in learning and memory area, one capability that gains much attention is the assessment of power of attention, which includes the abilities of attention and information processing [15]. This capability can be assessed by using response times in simple reaction time, choice reaction time, and digit vigilance task forces as indices because the response time reflects how the subjects pay attention to the stimuli. The more shorter response time indicates the better attention toward stimuli. Therefore, the present results indicate the increase in power of attention induced by anthocyanin-rich mulberry milk at both doses used in this study. In addition, the continuity of attention or the ability to maintain attention is also focused. It has been demonstrated that anthocyanin-rich mulberry milk also increases this ability manifested by the increase in percent accuracy in the performances of simple reaction time, choice reaction time, and digit vigilance tests. The computerized battery test can assess not only attention but also memory. Our data also reveal that subjects who consume anthocyanin-rich mulberry milk both at 1 serving and 2 servings per day significantly improve both response time and percent accuracy in word recognition, picture recognition, spatial, and numeric working memory. These changes indicate the improvement of quality and speed of memory. Therefore, our data reveal that anthocyanin-rich mulberry milk can improve 4 domains of working memory including attention, continuity of attention, speed of memory, and quality of memory.

Recently, it has been demonstrated that working memory shows a negative relationship with cortisol and AChE [8]. Therefore, we also investigate the effect of anthocyanin-rich mulberry milk on the mentioned parameters after 6 weeks of consumption period. Our data also confirm the effect of mulberry milk. It has been revealed that the saliva cortisol shows a significant decrease after a 6-week consumption period of anthocyanin-rich mulberry milk 1 serving per day. No change of AChE is observed. The significant reduction of both saliva cortisol and AChE is observed in subjects who consume an anthocyanin-rich mulberry milk 2 servings per day. In addition to the changes of the mentioned parameters, the significant reduction of MAO and MAO-A is also observed. It has been previously shown that the MAO and MAO-A are associated with working memory [16–20]. The suppression of MAO activity is reported to produce the improvement of memory impairment [20]. Due to these pieces of information, we do suggest that the enhancement of memory induced by an anthocyanin-rich mulberry milk may occur partly via the suppression of cortisol, AChE, MAO, and MAO-A. Based on the suppression effect of anthocyanin on AChE [21], MAO, and MAO-A [22], we suggest that the possible active ingredient may partly involve anthocyanin content in the mulberry milk.

Our data also demonstrate that subjects who consumed an anthocyanin-rich mulberry milk 2 servings daily also show the elevation of pulse rate, which indicates the elevation of heart rate, which in turn should enhance blood pressure. However, no significant change in blood pressure is observed. The possible underlying mechanism may involve the change of another parameter such as stroke volume, which also exerts an influence on blood pressure. Therefore, it is less likely to contribute a role on the improvement of working memory. In addition, the increased respiratory rate is also observed but still in the normal range. However, a recent study reveals that the increase in respiration can also pack more oxygen into hemoglobin, which in turn can enhance brain oxygen supply and brain function [23]. Due to this line of evidence, we also suggest that the increase in brain oxygen supply induced by the elevation of respiration may also play a role on the memory enhancement. A decrease in diastolic pressure is also observed in subjects, who consumed an anthocyanin-rich mulberry milk 1 serving daily. Since the most important factor that determines diastolic blood pressure is total peripheral resistance, we suggest that a reduction of diastolic blood pressure may possibly associate with the reduction of total peripheral resistance due to the reduction of arterial contraction induced by the reduction of sensitivity to vasoactive agents such as norepinephrine via the permissive action of cortisol [24]. This study failed to show the dose-dependent manner because the tested product contains many ingredients so the effect of active ingredient could be masked by the other ingredients.

## 5. Conclusion

This study demonstrates the memory enhancing effect of an anthocyanin-rich mulberry milk in working-age adults. The possible underlying mechanisms may occur primarily via the suppression of cortisol. The high dose can also exert the positive modulation effect via the suppression effect on AChE, MAO, and MAO-A. However, the increase brain oxygenation due to the increased respiration may also play a role and this point still requires further study to confirm it. The current data suggest that an anthocyanin-rich mulberry milk shows the potential to serve as the functional drink targeting at memory enhancement. Due to the suppression effect of cortisol, it may possibly exert the positive modulation effect on other stress-related disorders such as anxiety, depression, and insomnia but these points also require further investigation.

## Figures and Tables

**Table 1:** The characterization of subjects. Values are presented as mean ± SEM (*n* = 150/group). Comparison of data was performed between at baseline and at a 6-week study period. ^∗^*p* value < 0.05, ^∗∗^*p* value < 0.01, respectively.

Characteristics	Mulberry milk 1 serving/day		*p* value	Mulberry milk 2 servings/day		*p* value
	Baseline	6 weeks		Baseline	6 weeks	
Age (year)	36.39 ± 0.63	36.39 ± 0.63	1	34.84 ± 0.65	34.84 ± 0.65	1
Gender (male/female)	44/106	44/106		38/112	38/112	
PR (beats/min)	78.05 ± 0.90	79.78 ± 0.85	0.162	78.53 ± 0.97	81.34 ± 0.97	0.041^∗^
Respiratory rate (breaths/min)	17.50 ± 0.17	17.73 ± 0.12	0.256	17.09 ± 0.17	17.65 ± 0.13	0.009^∗∗^
Systolic BP (mmHg)	123.43 ± 1.08	122.37 ± 1.04	0.482	123.01 ± 1.14	122.01 ± 1.09	0.525
Diastolic BP (mmHg)	80.62 ± 0.80	78.05 ± 0.76	0.021^∗^	80.37 ± 0.83	78.59 ± 0.90	0.148
Body weight (kg)	61.63 ± 0.95	61.82 ± 0.96	0.893	63.28 ± 1.02	63.47 ± 1.00	0.895
Body height (cm)	160.70 ± 0.59	160.69 ± 0.59	0.987	161.12 ± 0.60	161.14 ± 0.60	0.988
Body mass index (kg/m^2^)	23.80 ± 0.31	23.87 ± 0.31	0.876	24.41 ± 0.39	24.48 ± 0.39	0.891

**Table 2:** Effect of mulberry milk consumption 1 serving per day on percent of accuracy response and response time of each cognitive assessment test. Values are presented as mean ± SEM (*n* = 150/group). Comparison of data was performed between at baseline and at a 6-week study period. ^∗^*p* value < 0.05, ^∗∗^*p* value <0.01, and ^∗∗∗^*p* value < 0.01, respectively.

Cognitive domains	Test items	Mulberry milk 1 serving/day		*p* value
Word recognition	Response time	Baseline	1304.81 ± 22.40	*p* < 0.001
		6 weeks	1136.83 ± 15.06^∗∗∗^	
	%accuracy	Baseline	84.12 ± 0.78	*p* < 0.001
		6 weeks	87.77 ± 0.66^∗∗∗^	
Picture recognition	Response time	Baseline	133.93 ± 24.56	*p* < 0.001
		6 weeks	1225.47 ± 18.06^∗∗∗^	
	%accuracy	Baseline	86.68 ± 0.62	*p* < 0.001
		6 weeks	90.07 ± 0.59^∗∗∗^	
Simple reaction	Response time	Baseline	650.95 ± 13.97	*p* < 0.001
		6 weeks	578.42 ± 9.74^∗∗∗^	
Digit vigilance	Response time	Baseline	623.68 ± 4.51	*p* = 0.477
		6 weeks	619.33 ± 4.13	
	%accuracy	Baseline	96.10 ± 0.24	*p* = 0.017
		6 weeks	69.98 ± 0.27^∗^	
Choice reaction time	Response time	Baseline	776.97 ± 0.21	*p* = 0.010
		6 weeks	745.54 ± 8.16^∗^	
	%accuracy	Baseline	97.90 ± 0.21	*p* = 0.534
		6 weeks	98.05 ± 0.14	
Spatial memory	Response time	Baseline	1405.60 ± 29.97	*p* < 0.001
		6 weeks	1269.92 ± 22.72^∗∗∗^	
	%accuracy	Baseline	90.76 ± 0.97	*p* = 0.002
		6 weeks	94.45 ± 0.63^∗∗^	
Numeric working memory	Response time	Baseline	11616.85 ± 16.63	*p* = 0.006
		6 weeks	1053.39 ± 15.93^∗∗^	
	%accuracy	Baseline	94.27 ± 0.76	*p* = 0.017
		6 weeks	96.49 ± 0.53^∗^	

**Table 3:** Effect of mulberry milk consumption 2 servings per day on percent of accuracy response and response time of each cognitive assessment test. Values are presented as mean ± SEM (*n* = 150/group). Comparison of data was performed between at baseline and at a 6-week study period. ^∗^*p* value < 0.05, ^∗∗^*p* value < 0.01, and ^∗∗∗^*p* value < 0.01, respectively.

Cognitive domains	Test items	Mulberry milk 2 servings/day		*p* value
Word recognition	Response time	Baseline	1335.46 ± 21.12	*p* < 0.001
		6 weeks	1143.61 ± 0.62^∗∗∗^	
	%accuracy	Baseline	83.57 ± 0.76	*p* < 0.001
		6 weeks	88.15 ± 0.62^∗∗∗^	
Picture recognition	Response time	Baseline	1336.74 ± 26.72	*p* = 0.006
		6 weeks	1246.17 ± 18.61^∗∗^	
	%accuracy	Baseline	87.28 ± 0.68	*p* < 0.001
		6 weeks	91.12 ± 0.68^∗∗∗^	
Simple reaction	Response time	Baseline	666.34 ± 17.29	*p* < 0.001
		6 weeks	577.41 ± 8.92^∗∗∗^	
Digit vigilance	Response time	Baseline	621.98 ± 4.34	*p* = 0.654
		6 weeks	619.31 ± 4.08	
	%accuracy	Baseline	95.93 ± 0.30	*p* = 0.002
		6 weeks	97.11 ± 0.23^∗∗^	
Choice reaction time	Response time	Baseline	779.86 ± 10.76	*p* = 0.004
		6 weeks	741.20 ± 7.88^∗∗^	
	%accuracy	Baseline	97.74 ± 0.18	*p* = 0.300
		6 weeks	97.99 ± 0.16	
Spatial memory	Response time	Baseline	1384.93 ± 27.99	*p* < 0.001
		6 weeks	1227.99 ± 19.34^∗∗∗^	
	%accuracy	Baseline	91.43 ± 1.09	*p* = 0.007
		6 weeks	94.87 ± 0.66^∗∗^	
Numeric working memory	Response time	Baseline	1098.42 ± 16.65	*p* = 0.008
		6 weeks	1039.73 ± 14.22^∗∗^	
	%accuracy	Baseline	95.73 ± 0.55	*p* = 0.334
		6 weeks	96.45 ± 0.51	

**Table 4:** The effect of mulberry milk 1 serving/day on cortisol level and the activities of AChE, MAO, MAO_A_, and MAO_B_ in saliva (*n* = 150/group). ^∗∗∗^*p* value < 0.001 compared to baseline.

Characteristics	Mulberry milk 1 serving/day		*p* value
	Baseline	6 weeks	
Cortisol level (ng/ml)	8.40 ± 0.12	6.94 ± 0.10^∗∗∗^	<0.001
AChE activity (nmol/mg protein)	13.09 ± 0.76	12.19 ± 0.58	0.347
MAO activity (*μ*mol/h/mg protein)	0.31 ± 0.02	0.29 ± 0.02	0.61
MAO_A_ activity (*μ*mol/h/mg protein)	0.16 ± 0.01	0.15 ± 0.01	0.705
MAO_B_ activity (*μ*mol/h/mg protein)	0.13 ± 0.01	0.13 ± 0.01	1

**Table 5:** The effect of mulberry milk 2 servings/day on cortisol level and the activities of AChE, MAO, MAO_A_, and MAO_B_ in saliva (*n* = 150/group) ^∗^*p* value < 0.05 and ^∗∗∗^*p* value < 0.001, respectively, compared to baseline.

Parameter	Mulberry milk 2 servings/day		*p* value
	Baseline	6 weeks	
Cortisol level (ng/ml)	8.20 ± 0.13	6.87 ± 0.09^∗∗∗^	<0.001
AChE activity (nmol/mg protein)	13.55 ± 0.52	12.02 ± 0.56^∗^	0.047
MAO activity (*μ*mol/h/mg protein)	0.32 ± 0.01	0.27 ± 0.01^∗^	0.013
MAO_A_ activity (*μ*mol/h/mg protein)	0.16 ± 0.01	0.14 ± 0.01^∗^	0.034
MAO_B_ activity (*μ*mol/h/mg protein)	0.15 ± 0.01	0.13 ± 0.01	0.126

## Data Availability

I confirm that data are available and will be provided on request.
